# Unveiling the Hidden Properties of Tomato Peels: Cutin Ester Derivatives as Bio-Based Plasticizers for Polylactic Acid

**DOI:** 10.3390/polym15081848

**Published:** 2023-04-12

**Authors:** Grazia Isa C. Righetti, Rita Nasti, Giangiacomo Beretta, Marinella Levi, Stefano Turri, Raffaella Suriano

**Affiliations:** 1 Department of Chemistry, Materials and Chemical Engineering “Giulio Natta”, Politecnico di Milano, Piazza Leonardo da Vinci 32, 20133 Milano, Italy; 2 Department of Environmental Science and Policy, Università degli Studi di Milano, Via Celoria 2, 20133 Milano, Italy; 3 National Interuniversity Consortium of Materials Science and Technology, Via G. Giusti 9, 50121 Firenze, Italy

**Keywords:** bio-based polymers, tomato peels, biomass valorization, agricultural waste, cutin, fatty acids, plasticizers, PLA, bioplastics

## Abstract

Polylactic acid (PLA) is one of the most important biopolymers employed on the market due to its good mechanical strength and barrier properties. On the other hand, this material presents a rather low flexibility, limiting its employment. The valorization of bio-based agro-food waste for the modification of bioplastics is a highly appealing approach for the replacement of petrol-based materials. The aim of this work is to employ cutin fatty acids derived from a biopolymer (i.e., cutin), present in waste tomato peels and its bio-based derivatives as new plasticizers to enhance PLA flexibility. In particular, pure 10,16-dihydroxy hexadecanoic acid was extracted and isolated from tomato peels and then functionalized to give the desired compounds. All the molecules developed in this study were characterized by NMR and ESI-MS. Blends at different concentrations (10, 20, 30, and 40% *w*/*w*) the flexibility (T_g_ measurements with differential scanning calorimetry—DSC) of the final material. Furthermore, the physical behavior of two blends obtained by mechanical mixing of PLA and 16-methoxy,16-oxohexadecane-1,7-diyl diacetate was investigated through thermal and tensile tests. The data collected by DSC show a lowering in the T_g_ of all the blends of PLA with functionalized fatty acids, in comparison with pure PLA. Lastly, the tensile tests highlighted how PLA blended with 16-methoxy,16-oxohexadecane-1,7-diyl diacetate (20% *w*/*w*) can efficiently enhance its flexibility.

## 1. Introduction

Over the last two decades, public awareness about increasing environmental pollution and the limited reserves of fossil fuel has been constantly rising, leading human beings to realize how our current civilization is almost unsustainable [1]. The cause of the current concern regarding climate change and resource limitations can be traced back to the industrial revolution and the related economic and population growth which resulted in a higher demand for new chemicals and materials [2]. It is a proven fact that most of our common goods and manufacturing products derive from petroleum feedstock. Nowadays, one of the major challenges that researchers are facing is related to a shift to a greener and more sustainable approach to producing chemicals and materials. Under this perspective, the valorization of biomass as a renewable source for the production of energy, chemicals, and material is the key to achieving sustainable development [3]. Among materials obtained from biomass, bio-derived polymers, often also called bioplastics, stand out as an exemplary case, whose development can lead to a big step toward the realization of a circular economy [4].

Although petrol-based synthetic polymers are unique materials due to their versatility, their physical, chemical, and mechanical properties as well as the ease of production and processability [5], they intrinsically lack sustainability due to their derivation from non-renewable feedstocks and their non-biodegradability in a medium to a short period of time [6]. This triggered the development of new bio-based and biodegradable polymers. Polylactic acid (PLA) is one of the most used biodegradable and renewable polyesters on the market. Due to its peculiar properties, such as high tensile strength and biocompatibility, it emerges as one of the leading candidates to replace petrol-based polymers [7]. Despite all the positive features, PLA still has some disadvantages that can limit its application in sectors including packaging, such as its brittleness at room temperature, high glass transition temperature, poor water-barrier properties [7], and the lack of reactive side chain that can make its modification quite challenging [8]. In particular, PLA needs to be plasticized to improve its flexibility and ductility in ambient conditions and to be successfully employed as a food packaging film [9]. To achieve better flexibility and toughness, different attempts have been made by: (i) the incorporation of lactic acid oligomers [10]; (ii) the introduction of small molecular weight substances, e.g., glycerol and its derivates, vegetable oil derivates [11,12], adipates [13] and citrates [14]; and (iii) the blending with other biodegradable polymers and copolymers [15]. Of these solutions, some presented bio-based plasticizers for PLA are derived from vegetable oils [16,17,18], to further reduce the environmental impact and potentially minimize the risks related to the toxicity of conventional additives. In the framework of the circular economy model, bio-based PLA plasticizers can be also obtained from agricultural resources, such as sunflower oil [17], palm oil, and soybean oil [18], and from agro-industrial residues, i.e., liquefied wood flour esters, which showed good miscibility with PLA and high plasticization efficiency [19].

The amount of agricultural waste deriving from harvestable lands is a real issue as most of the environmental impact related to this sector is strictly connected to food loss and waste [20,21]. In this context, the valorization of food waste really fits in the perspective of tackling the mentioned issues as it represents a cheap, abundant, and renewable source [22] rich in many useful organic substances that could be isolated and re-employed [23]. Among all, cutin is a highly interesting and abundant plant-derived biopolymer, present in plant cuticles, such as tomato skins [24]. It is a non-toxic biodegradable crosslinked polyester whose main components are polyhydroxylated C_16_ and C_18_ fatty acids [25]. Considering its structural properties, several research works took it in consideration to develop new plant cuticle-inspired polyesters as coating layers for food cans [26] and hydrophobic films for packaging applications [27,28,29], as well as for the production of antimicrobial cutin oligomers [30]. On the other hand, due to its structural features, cutin monomer could also be employed as a plasticizing agent for polymeric mixtures; up to now, to the best of our knowledge, no studies of this kind have yet been reported in the literature.

With this work, we aim at improving PLA flexibility by employing 10,16-dihydroxyhexadecanoic acid (10,16-diHHDA)—the main cutin monomer—and its derivatives as bio-based plasticizer agents. This component was extracted from tomato peels with a good purity degree and then functionalized by different esterification and acetylation reactions. The pristine cutin-derived fatty acid and its derivatives were then characterized, and their plasticizing effect was analyzed. The presence of ester groups in cutin derivatives can in fact form diverse interactions with PLA’s ester groups, improving the miscibility between the modified monomers and the polyester. Moreover, the flexible alkylic chains can positively intercalate between PLA chains, thus increasing intermolecular spacing and bringing mobility and a good plasticizing effect [9]. Blends of PLA and 10,16-diHHDA derivatives also showed a clear decrease in the glass transition temperature by differential scanning calorimetry (DSC), resulting in good plasticization efficiency and an evident increase in ductility by tensile tests. This work, therefore, can pave the way for the use of cutin fatty acid derivatives as bio-based plasticizers for other bioplastics.

## 2. Materials and Methods

### 2.1. Materials

All reagents and solvents were purchased from Merck (Merck Life Science S.p.A., Milan, Italy) and used without any further purification if not otherwise specified. The 10,16-diHHDA (**1**) was isolated from the tomato peel supplied by TomatoFarm s.r.l. (Bettole di Pozzollo (AL), Italy); PLA (Ingeo Biopolymer 2003D) was purchased from NatureWorks LLC (Nebraska, USA).

### 2.2. Synthesis Procedures

#### 2.2.1. 10,16-Dihydroxy Hexadecanoic Acid Isolation (10,16-diHHDA, **1**)

An amount of 30.12 g of degreased tomato peels were placed in a one-neck round bottom flask equipped with a magnetic stirrer. An amount of 300 mL of a 1 M solution of NaOH in methanol were added to the flask obtaining a red suspension which is kept at 90 °C for 2.5 h. The suspension was cooled at r.t., filtered on Büchner, and washed with distilled water (600 mL). The red solution (pH~13) was acidified to pH ~ 3, so that a yellow solid precipitate was visible while the acidification occurred. The suspension was extracted with dichloromethane (200 mL × 5), then the solvent was removed at the rotavapor to make the product a yellow waxy solid (6.82 g, 23.6 mmol, 20% yield, 92% purity).

^1^H-NMR (CDCl_3_) δ (ppm): 1.1–1.6 (br. m., 26H, aliphatic CH_2_), 2.28 (t, 2H, *J* = 7.7 Hz, -CH_2_-COOH), 3.52 (br. s – -OH), 3.57 (t, 2H, *J* = 6.5 Hz, -CH_2_-OH), 3.65 (q, 1H, *J* = 7.06, 14.02 Hz, -CH(OH)-)

^13^C-NMR in CDCl_3_ δ (ppm): 178.57, 71.98, 63.09, 37.32, 37.26, 29.44, 29.37, 29.22, 29.03, 28.99, 25.67, 25.52, 25.46, 24.65

GC-MS: R*t*: 33.52 min

+MS (ESI) (methanol): *m/z* 289.3 [M + H]^+^, 311.3 [M + Na]^+^

#### 2.2.2. Methyl-10,16-dihydroxy Hexadecanoate Synthesis (10,16-diHHDME, **2**)

500.0 mg (1.56 mmol) of **1** were placed in a round bottom flask, then 5 mL of methanol and 250 µL of HCl 1 M were added, obtaining a yellow solution which was kept under stirring at 50 °C for 16 h. The solvent was removed under reduced pressure, obtaining a brown oil. This was dissolved in 10 mL of diethyl acetate and extracted with a 0.1 M solution of NaCl (3 × 15 mL). The solution was dried on sodium sulfate and the solvent was removed, obtaining the compound as a brown oil (88 % yield, 90% purity).

^1^H-NMR in CDCl_3_ δ (ppm): 1.1–1.5 (br. m., 26H, aliphatic CH_2_), 2.23 (t, 2H, *J* = 7.7 Hz, -CH_2_-COOH), 3.50 (br. m, 1H, -CH(OH)-), 3.55 (t, 2H, *J* = 6.55 Hz, -CH_2_-OH), 3.6 (s, 3H, -COOCH_3_)

^13^C-NMR in CDCl_3_ δ (ppm): 174.3, 72.88, 62.97, 51.35, 37.47, 37.34, 34.08, 32.65, 29.57, 29.43, 29.36, 29.16, 29.09, 28.61, 27.70, 25.57, 24.92

+MS (ESI) (methanol): *m/z* 285.2 [M + H – H_2_O]^+^, 303.2 [M + H]^+^, 573.6 [2·M – OCH_3_ + 2H]^+^ (protonated dimer)

#### 2.2.3. 10,16-Diacetoxy Hexadecanoic Acid Synthesis (10,16-diAHDA, **3**)

500.0 mg (1.56 mmol) of **1** were placed in a round bottom flask, then 5 mL of acetic anhydride Ac_2_O and 25 µL of concentrated HCl were added. The mixture, a light brown solution, was kept under stirring at 100 °C for 4 h. The mixture was cooled to room temperature and the solvent was removed, obtaining a brown oil that is recovered with 10 mL of diethyl acetate and extracted with a 0.1 M solution of NaCl (3 × 15 mL). The solution was dried on sodium sulfate and the solvent is removed, obtaining the compound as a brown oil (88% yield, 73% purity).

^1^H-NMR (CDCl_3_) δ (ppm): 1.1–1.6 (br. m., 26H, aliphatic CH_2_), 1.96–1.97 (overlapping singlets, 6H, -COOCH_3_) 2.37 (t, 2H, *J* = 7.7 Hz, -CH_2_-COOH), 3.9 (t, 2H, *J* = 6.9 Hz, -CH_2_-OAc) 4.77 (q, 1H, *J* = 6.2, 12.5 -CH(OAc)-)

^13^C-NMR in CDCl_3_ δ (ppm): 179.26, 171.29, 171.02, 74.32, 64.56, 34.10, 33.93, 29.41, 29.37, 29.25, 29.13, 29.09, 28.99, 28.50, 25.80, 25.25, 25.18, 24.64, 21.26, 20.98 

+MS (ESI) (methanol): *m/z*, 373 [M + H]^+^, 395 [M + Na]^+^

#### 2.2.4. 16-Methoxy-16-oxo-hexadecane-1,7-diyl Diacetate Synthesis (10,16-diAHDME, **4**)

250 mg of **2** were placed in a round bottom flask, and 2.5 mL of Ac_2_O and 25 µL of concentrated HCl were added. The resulting light brown solution is heated at 100 °C for 1 hour. The solvent is removed under reduced pressure and the product is obtained as a brown oil (92% yield, 83% purity). 

^1^H-NMR (CDCl_3_) δ (ppm): 1.1–1.5 (br. m., 26H, aliphatic CH_2_), 1.7–1.9 (s, 6H, -COCH_3_), 2.22 (t, 2H, *J* = 7.4 Hz, CH_2_-COOCH_3_), 3.5 (s, 3H, -COOCH_3_), 3.9 (t, 2H, *J* = 6.9 Hz, -CH_2_-OAc), 4.7 (q, 1H, *J* = 6.2, 12.5 Hz, -CH(OAc)-) 

^13^C-NMR in CDCl_3_ δ (ppm): 174.17, 171.07, 170.81, 74.18, 64.45, 51.34, 34.07, 34.02, 34.00, 29.38, 29.24, 29.09, 29.04, 28.49, 25.78, 25.23, 25.15, 24.87, 21.19, 20.91 

+MS (ESI) (methanol): *m/z* 409.4 [M + Na]^+^

#### 2.2.5. 16-Methoxy-16-oxo-hexadecane-1,7-diyl Diacetate (**4**) from 10,16-Dihydroxy Hexadecanoic Acid Synthesis (**1**)—Telescoped Reaction

500 mg of **1** were placed in a round bottom flask together with 5 mL of methanol and 250 µL of concentrated HCl. The system, an orange solution, was kept at 40 °C for 18 h, then it was brought back to room temperature. The solvent was evaporated under reduced pressure. An amount of 10 mL of acetic anhydride and 100 µL of concentrated HCl were added to the system, which was then heated at 100 °C for 90 min. The solvent was then removed at the rotary evaporator obtaining the final product as a dark brown oil (76% yield, 78% purity). 

All the cutin derivatives need to be stored under nitrogen and at low temperatures or to be used right after preparation.

### 2.3. Blend Preparation Procedures

#### 2.3.1. Solution Blending—Procedure for Monomer **1**

Monomer **1** was firstly dissolved in 30 mL of chloroform under heating to give a yellow solution. PLA was then added and the mixture was left under stirring at room temperature until a homogeneous yellow mixture was obtained (3 h). This is poured into a Petri dish and left firstly under the fume hood for 48 h, then in the vacuum oven at 60 °C for 2 h to remove any residues of chloroform. The final film was collected and analysed with DSC.

#### 2.3.2. Solution Blending—General Procedure for **2**, **3**, and **4**

The derivative was firstly dissolved in 30 mL of chloroform (**2**, **3**, and **4** at room temperature), then PLA was added and the mixture was left under stirring at room temperature until a homogeneous light brown mixture was obtained. This was poured into a Petri dish and left firstly under the fume hood for 48 h, then in the vacuum oven at 60 °C for 2 h to remove any residues of chloroform. The final film is collected and analysed with DSC.

#### 2.3.3. Mechanical Melt Blending—Preparation of PLA + 16-Methoxy-16-oxo-hexadecane-1,7-diyl Diacetate (**4**)

PLA was melted on a heating plate at 190 °C, cut into small pieces, and then cooled down in ice for 10 min to solidify the polymer and reduce the crystallinity degree, allowing a faster melting in the following mixing step. The pieces were then placed in a mixer, cut to a smaller size, and placed in a vacuum oven at 50 °C for 3 h to remove any traces of water. The PLA was then placed inside an internal mixer (Brabender^®^) at 180 °C with a rotating speed of 30 rpm, then monomer **4** is slowly added to the mixer over 20 min. Afterward, the mixture was left under mixing for another 10 min. The final mixture is collected as a brown homogeneous solid.

### 2.4. Characterization Techniques

The ^1^H-NMR and ^13^C-NMR spectra were recorded on a Bruker AV 400 MHz instrument (Bruker Corporation, Billerica, MA, USA), equipped with a 5 mm multinuclear probe. The ^1^H-NMR analysis for the analytical yield and purity was performed in the presence of 1,4-dinitro benzene as an external standard. Mass spectra were recorded by using Electrospray Ionization (ESI) with a Bruker Esquire 3000 (Bruker Corporation, Billerica, MA, USA) plus ion-trap mass spectrometer instrument equipped with an ESI Ion Trap LC/MSn System, injecting in the instrument a diluted solution of each sample as follows: 1 mg of sample in 1 mL of dichloromethane (DCM).

The purity of 10,16-diHHDA was determined by Bruker Scion SQ instrument (Bruker, Milan, Italy) gas chromatography equipped with a ZB-5HT INFERNO capillary column (30 m; 0.25 mm i.d., film thickness 0.25 mm) (Phenomenex s.r.l., Castel Maggiore (BO), Italy). The oven temperature was initially set at 125 °C (hold time 3 min), with a gradient from 125 to 205 °C (5.0 °C/min, hold 7 min), and from 205 to 300 °C (7 °C/min, hold 5 min). The following operation parameters were then used: injector temperature = 300 °C; column flow = 1.00 mL/min; carrier gas helium = 5.5; ionization energy = 70 eV. The split/splitless ratio was set to 1:30 after 75 s. An aliquot of 10,16-diHHDA was derivatized by the addition of 30 μL of pyridine and 70 μL of N,O-bis(trimethylsilyl)trifluoroacetamide (BSTFA). After 1.5 h of incubation at 62 °C, 500 μL of ethyl acetate was added and 2.0 μL of the solution was injected into the GC-MS system. Identification was executed by comparison with the retention times and fragmentation patterns of authentic standards when available, or by comparison with those present in the National Institute of Standards and Technology (NIST) spectral library (NIST, 2011 vers. 2.0), or by comparison with those present in the literature) [31].

Differential scanning calorimetry (DSC) was performed with a DSC 823e (Mettler-Toledo, Columbus, OH, USA). All runs were performed on 5–20 mg samples under a nitrogen atmosphere. The data obtained were used to measure characteristic temperatures and enthalpies. The DSC thermal history consisted of: (i) first heating run from −100 °C to 200 °C (20 °C min^−1^); (ii) cooling step from 200 °C to −100 °C (20 °C min^−1^); (iii) second heating run from −100 °C to 200 °C (20 °C min^−1^). The T_g_ was determined as the inflection point of DSC profiles during the second heating run while the T_m_ is the melting peak temperature present in the first heating run. The crystallinity degree (%) was calculated using the following equation:

(1) $$\frac{\Delta H_{melt}-{\Delta H}_{cryst}}{{\Delta H}_{melt,th}*w_{PLA}}*100$$

where $\Delta H_{melt}$ is the melting enthalpy, ${\Delta H}_{cryst}$ is the crystallization enthalpy which are both calculated as the area underneath the melting and crystallization peaks, respectively, in the first DSC heating scan, ${\Delta H}_{melt,th}$ is the theoretical melting enthalpy for a 100% crystalline PLA that has a value of 93 (J/g), as reported in the literature [32] and $w_{PLA}$ is the weight fraction of plasticized PLA.

PLA tensile properties were determined at room temperature using a Zwick/Roell Z010 (Zwick Roell Italia, Genova, Italy) equipped with a 10 kN load cell and a long-stroke extensometer. The tensile test was performed on dumbbell specimens having a gauge length of 22 mm at a rate of 50 mm/min, following the standard test method reported by ASTM International [33]. The specimens were manufactured by injection molding using a Babyplast 6/10 horizontal model. Young’s modulus was determined as the slope of the line tangent to the stress-strain curves at strain values of 0.2%, while the toughness values were calculated as the area underneath the stress-strain curves over the entire range of measured strain values. The cross-sectional areas obtained at the end of tensile tests at the break of dumbbell specimens were covered with a thin metallic gold film and were analysed by scanning electron microscopy (SEM) using an extended pressure SEM Zeiss EVO 50 EP microscope (Zeiss, Oberkochen, Germany).

## 3. Results and Discussion

To improve PLA flexibility and its mechanical toughness, cutin-derived fatty acid and its derivatives were tested as plasticizing agents.

**Scheme 1:**
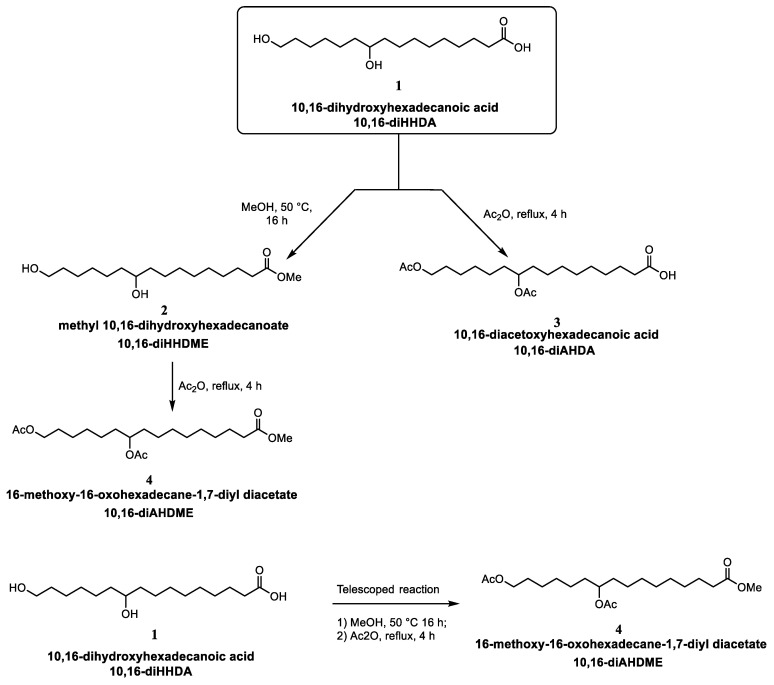
Synthesis of 10,16-di HHDA (**1**) derivatives (**2**–**4**). Protection of the hydroxyl function of **1** in the presence of Ac_2_O gives rise to derivative **3**. Esterification of the acidic function of **1** brings to molecule **2**, which is a precursor of **4**. A telescoped reaction can also be performed, allowing to obtain molecule **4** directly from **1** without the need to isolate the intermediate **3**.

**Figure A1:**
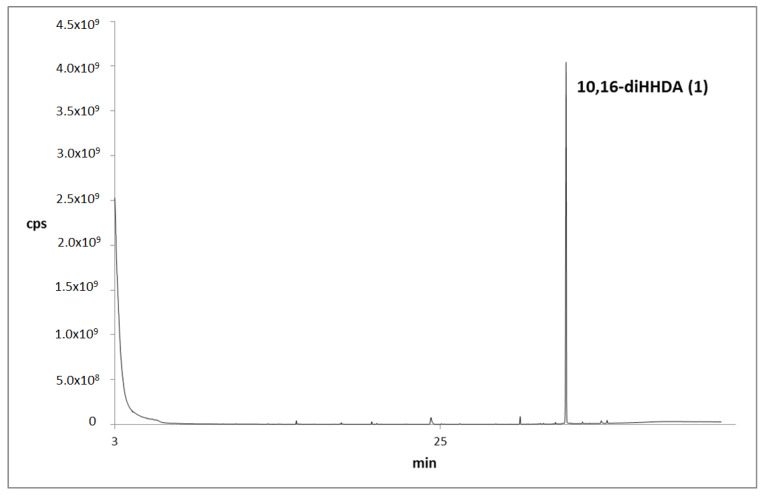
Chromatographic profile obtained by performing a gas chromatographic-mass spectroscopy (GC-MS) analysis of cutin monomer **1** after the depolymerization reaction.

From degreased tomato peels, 10,16-diHHDA (**1**) was isolated by methanolysis according to Osman et al. [34] with some modifications. The crude resulted in a waxy yellowish solid with a purity of 95%, as confirmed by gas chromatographic-mass spectroscopy (GC-MS) analysis (Figure A1) and then functionalized to give the corresponding ester derivatives (**2**–**4**, Scheme 1).

10,16-Dihydroxy hexadecanoic methylester (10,16-diHHDME, **2**) was synthesized by reacting **1** with methanol in an acidic environment, obtaining as a result the desired product in medium to high yields (78–90%). The acetylation was performed under different conditions to find the best protocol (Table A1). When the reaction is carried out in the presence of an excess of acetic anhydride and using concentrated HCl as the catalyst, the reaction brings the formation of the final product in 4 hours at 100 °C (78–88% yield).

Additionally, a telescoped reaction can be efficiently conducted to obtain **4** starting from cutin monomer **1** without the need to isolate intermediate **2**.

**Figure 1:**
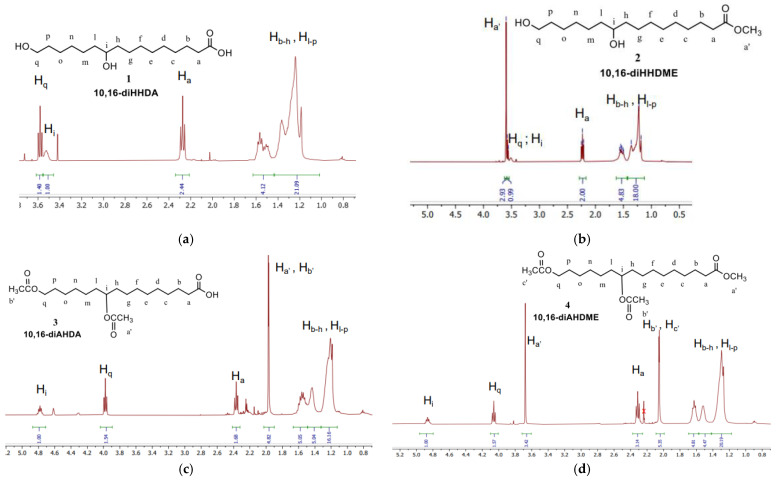
^1^H-NMR spectra in CDCl_3_ of the synthesized compounds (**1**–**4**): (**a**) ^1^H-NMR of 10,16-diHHDA (**1**); (**b**) ^1^H-NMR of 10,16-diHHDME (**2**); (**c**) ^1^H-NMR of 10,16-diAHDA (**3**); (**d**) ^1^H-NMR of 10,16-diAHDME (**4**).

The formation of the desired compounds was confirmed by ^1^H-NMR and MS analysis. The ^1^H-NMR of the isolated products with the assignment is reported in Figure 1. The presence of a singlet at 3.6 ppm (Figure 1b vs. Figure 1a) confirms the formation of the methyl ester **2**, while a shift of H_i_ and H_q_ positions to 4.77 ppm and 3.9 ppm together with the appearance of two overlapping singlets at 1.96 and 1.97 ppm indicates the obtaining of the diacetylated monomer **3** (Figure 1c vs. Figure 1a). Lastly, the combination of the shifts of the protons H_i_ and H_q_ together with the appearance of the singlets related to the methyl ester and the acetoxy group indicate the formation of the difunctionalized monomer **4** (Figure 1d vs. Figure 1a). For all the compounds, the methylene groups of the aliphatic chain appear as broad signals between 1.2 and 1.7 ppm.

The 10,16-diHHDA isolated has an experimental melting temperature of 59 °C (Table 1, **entry 1**), which is lower than the one reported in the literature (74–75 °C) [35]. This difference might be due to the different extraction and isolation conditions from the raw material. The melting temperature of methyl ester **2** is 12 °C and those of the other monomers are below zero (**entries 2**–**4**). These latter and the low T_g_ values reported for the monomers (**entries 2**–**4**) are in agreement with their physical state as they can all be isolated as brown oils.

### 3.1. Hansen Solubility Parameter Analysis

To estimate the compatibility of the synthesized derivatives of the cutin monomer with PLA, the solubility parameter, $\delta_{t}$, was calculated as follows:

(2) $$\delta_{t}=\sqrt{\delta_{d}^{2}+\delta_{p}^{2}+\delta_{h}^{2}}$$

where $\delta_{d}$, $\delta_{p}$, $\delta_{h}$ are, respectively, the dispersion, polar, and hydrogen bonding components of the solubility parameters that were evaluated by the Hoftyzer-Van Krevelen group as follows: contribution method [36].

(3) $$\delta_{d}=\frac{\sum \;F_{di}}{V}$$

(4) $$\delta_{p}=\frac{\sqrt{\sum \;F_{pi}^{2}}}{V}$$

(5) $$\delta_{h}=\sqrt{\frac{\sum \;E_{hi}^{}}{V}}$$

The dispersive, polar, and hydrogen bonding solubility parameter components ($\delta_{d}$, $\delta_{p},$ $\delta_{h}$) were calculated using Equations (2)–(5) for all the molecules **1**–**4**, using the dispersion components $F_{di}$ and the polar components $F_{pi}$ of the molar attraction constant as well as the hydrogen bonding energies $E_{hi}^{}$ for each functional group *i* (Table A2) [36]. The molar volume *V* was calculated by means of values of increments in Hoy’s System for the molar attraction function, according to the procedure explained in Appendix A. The computed solubility parameters and their components were compared with the corresponding values of PLA already present in the literature (Table 2) [37].

The contribution given by the dispersion forces ($\delta_{d}$—**entry 1**, Table 2) remains quite stable despite the functionalization and the difference from the dispersive value reported for PLA is around 2.5. The gap in the polar terms ($\delta_{p}$—**entry 2**) between the monomers and PLA is narrower than the values of **entry 1**, for 10,16-diHHDA (**1**, Scheme 1) and 10,16-diAHDME (**4**) being the compounds with the $\delta_{p}$ contribution closest to the PLA. The hydrogen-bonding contribution (**entry 3**) decreases significantly with the increasing functionalization of **1**, with monomer **4** having the closest value to the PLA. This result is in line with expectations about the effect of the esterification of the carboxylic acid and the protection of the two hydroxyl functional groups originally present on monomer **1**. These two functionalizations, in fact, were performed to reduce the possibility of the difunctionalized monomer (**4**) creating hydrogen bonding. The 16-methoxy,16-oxohexadecane-1,7-diyl diacetate (**4**) is, therefore, the molecule that presents the closest values of $\delta_{p}$ and $\delta_{h}$ to PLA and it is the one that is expected to give the best blending result with polylactic acid.

### 3.2. Solution Blending

PLA was blended in different concentrations with monomers **1**–**4** (Scheme 1) and the properties of the corresponding mixture were tested to evaluate the changes when compared to pure PLA. The maximum concentration achieved when blending compound **1** with PLA resulted in 20% *w*/*w*. After that, macroscopically non-homogeneous mixtures were formed. This result can be ascribed to the low solubility of 10,16-diHHDA in chloroform even when heating (at a temperature lower than the boiling temperature for CHCl_3_); for the derivatives **2**–**4**, higher concentrations could be obtained and the chosen ones were 20%, 30%, and 40% *w*/*w*.

From here on, the mixtures will be referred to as PLA−X−Y where X stands for the molecule (**1**–**4**) employed and Y for its concentration in the blending.

**Figure 2:**
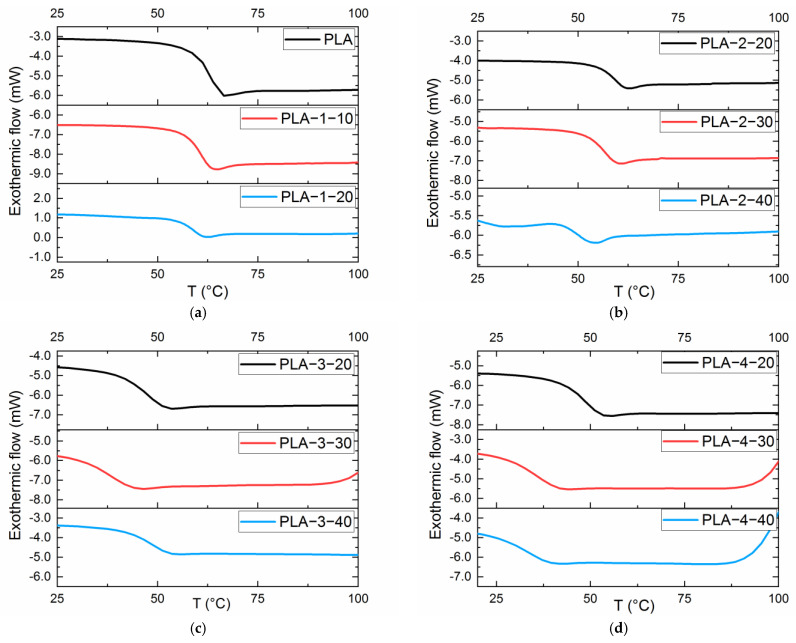
DSC profiles showing the glass transition temperatures evaluated as the inflection point during the second heating step (**a**) for pristine PLA and mixtures PLA−1−10 and PLA−1−20 with 10 and 20% *w*/*w* of cutin monomer **1** in the blending with PLA; and (**b**–**d**) for mixtures PLA−X−Y where X stands for the molecule (**2**–**4**) employed and Y for its concentration in the blending with PLA.

The plasticizing effect of the synthesized molecules on PLA was investigated through calorimetric tests (Table 3). The glass transition temperature (T_g_) resulted in being lower for all the blends than for the neat PLA polymer (Table 3, **entry 1**), indicating a softer and more ductile material. As also shown in Figure 2, the glass transitions appeared to be well-marked and steep for most of the mixtures under investigation, covering a temperature range of ≈10–20 °C from the onset to the end of the transition. As a general trend, a higher concentration of the monomers in the mixture usually led to a greater lowering of the glass transition temperature. By comparing blends with the same concentration (i.e., 30% *w*/*w*) but different plasticizers, it can be seen how the disubstituted molecule 10,16-diAHDME (**4**) is the one that leads to a greater change in terms of chain mobility and therefore material flexibility, with T_g_ passing from 57 °C to 36 °C (**entries 5**, **8**, **11**). This result is in agreement with the free volume theory [9], considering that the steric hindrance introduced by the substitution of the hydroxyl groups with the acetoxy and by the esterification of the acidic functional group of cutin monomer **1** induces an increase in the free volume, resulting in enhanced mobility of the polymeric chains that soften the final material.

**Figure A2:**
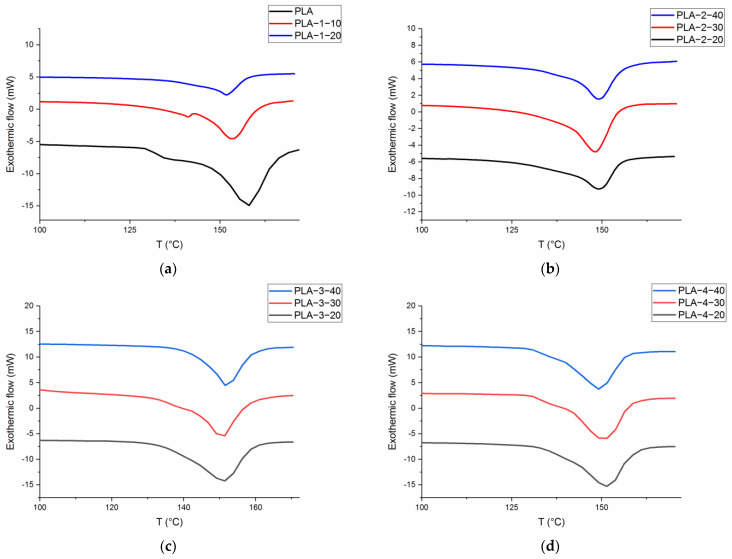
DSC profiles corresponding to the temperature regions with the PLA melting during the first heating run (**a**) for pristine PLA and mixtures PLA−1−10 and PLA−1−20 with 10 and 20% w/w of cutin monomer **1** in the blending with PLA; and (**b**–**d**) for mixtures PLA−X−Y where X stands for the molecule (**2**–**4**) employed and Y for its concentration in the blending with PLA.

The addition of molecules **2**–**4** did not seem to have a significant effect on the temperatures and shapes of melting peaks of plasticized PLA, compared to neat PLA (Figure A2 and Table 3). A little increase in the crystallinity degrees is also evident by increasing the concentration of the derivatives **2**–**4**, which facilitated the PLA crystallization (Table 3). On the other hand, cutin monomer **1** induced a little decrease in T_m_ (Figure A2, PLA−1−10 and PLA−1−20) and a clear reduction in crystallinity degree compared to pristine PLA (Table 3).

### 3.3. Mechanical Properties

The effect of the cutin-derived molecules on the mechanical properties of PLA was investigated by tensile testing and 16-methoxy,16-oxohexadecane-1,7-diyl diacetate (**4**, Scheme 1) was chosen as the additive for the blending with PLA for two reasons:

1. The results of the solubility parameter calculations, which highlighted how molecule **4** is the one with $\delta_{p}$ and $\delta_{h}$ values closest to the ones of PLA;
2. When compared to the other monomer derivatives, compound **4** showed a higher plasticizing effect with a greater reduction in T_g_.

**Figure 3:**
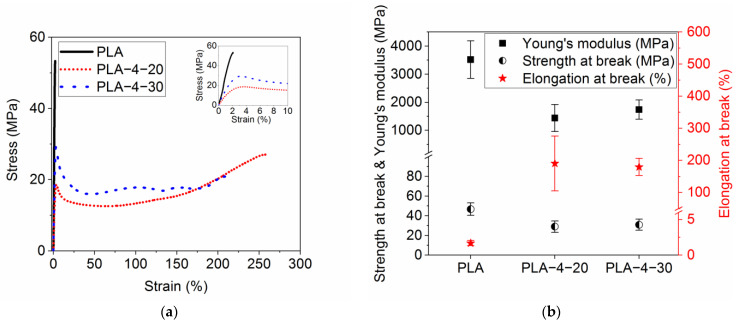
(**a**) Stress vs. strain curves obtained for representative samples of pristine PLA (black solid line), PLA with 20% w/w of cutin derivative **4** (PLA−4−20, red short dotted line), and PLA with 30% *w*/*w* of cutin derivative **4** (PLA−4−30, blue dotted line), inset: zoom-in of the stress-strain curves with a 0–10% strain range; (**b**) Comparison of Young’s modulus (squares), strength at break (circles) and elongation at break (stars) for pristine PLA, PLA−4−20, and PLA−4−30 and the error bars represent the standard deviations of average values.

For tensile tests, PLA−4−20 and PLA−4−30 were selected as promising samples since PLA−4−40 did not show any further reduction in T_g_ compared to PLA−4−30 and required a higher amount of plasticizer (**entries 10**–**12**, Table 3). Figure 3a presents the stress vs. strain curves obtained for representative samples of pristine PLA, PLA−4−20, and PLA−4−30 by tensile testing. The tensile curves highlighted how the ductility of PLA in the presence of molecule 4 was strongly enhanced (Figure 3) with an elongation at break of 190–200% in comparison with the neat PLA with a maximum deformation of 2–3%. Moreover, a slight decrease in Young’s modulus was also observed, considering that the elastic modulus for PLA−4−20 and PLA−4−30 is, respectively, 2.5 and 2 times lower than for pristine PLA (Figure 3b). The introduction of cutin derivative **4** also induced a small decrease in the tensile strength at break. However, such results in terms of tensile strength and strain at break values are comparable with those obtained for PLA plasticized with 10% and 30% *w*/*w* of a liquefied wood flour-derived ester [19] obtained from agro-industrial residues. Other studies concerning the plasticization of PLA, i.e., with sunflower-oil biodiesel, reported nearly 15% of elongation at break with 20% *w*/*w* of the additive [17], a lower value than those measured in this study. Therefore, these findings demonstrate how cutin derivative **4** can efficiently act as a plasticizer, making PLA more ductile and flexible, in agreement with the plasticization effect evidenced by DCS results.

**Figure 4:**
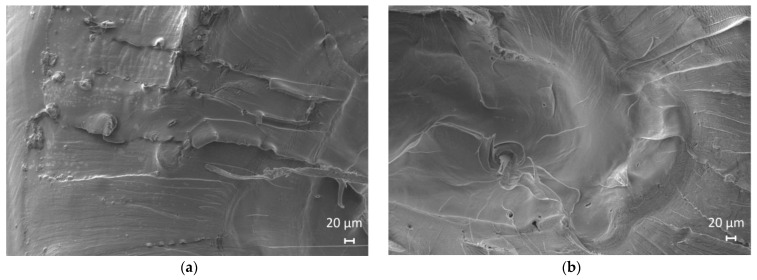
Scanning electron microscopy (SEM) images obtained examining the cross-sections induced at the end of tensile tests at the break of dumbbell specimens for (**a**) PLA−4−20 and (**b**) PLA−4−30.

To assess the presence of any phase separation of derivative **4** in the PLA matrix, SEM analyses were performed for PLA−4−20 and PLA−4−30. Figure 4 shows the SEM images of cross-sectional areas induced at the end of tensile tests at the break of dumbbell specimens. In the case of PLA−4−20, some 20 μm wide spots, brighter than the rest of the cross-section, can be identified (Figure 4a) and could indicate the occurrence of potential plasticizer segregation. However, no evidence of phase segregation was observed in Figure 4b and also in other regions for PLA−4−30, suggesting good compatibility between PLA and derivative **4** was achieved even at a higher concentration of plasticizer.

In terms of toughness, the value measured for pristine PLA is 63 ± 17 (N·mm) which is in line with the brittleness of the material. On the contrary, the mixture PLA−4−20 and the mixture PLA−4−30 present, respectively, a value of 7437 ± 1314 (N·mm) and 6999 ± 1045 (N·mm). These results highlight once more how the cutin derivative **4** can enhance the toughness and the capability of PLA to undergo plastic deformation. 

## 4. Conclusions

The cutin main monomer, i.e., 10,16-dihydroxy hexadecanoic acid, was isolated from the cutin present in tomato peels and then functionalized to give three different derivatives in medium to high yields. The isolated and functionalized molecules were characterized by NMR spectroscopy. The possibility of employing the cutin-derived fatty acid and its derivatives as plasticizing agents for PLA was tested.

The four cutin-derived molecules were blended with PLA in different concentrations (20%, 30%, and 40% w/w) and they all brought a lowering of the T_g_ of the final material. DSC analyses showed that a more evident effect on T_g_ was observed using the cutin derivative **3**, where the only substitution of hydroxyl groups of cutin monomer with acetoxy groups was performed, and using **4**, where the esterification of the acidic function of cutin acid was also carried out. The derivative **4** was selected as a more promising plasticizer since its solubility parameters were estimated to be closer to that of PLA.

An optimization in the material flexibility was achieved when 20% and 30% *w*/*w* of derivative **4** were introduced into the PLA matrix, considering the decrease of 15 and 30 °C in T_g_, respectively, and the increase of 100 times in the elongation at break and toughness values measured for the plasticized PLA, compared to the pristine PLA. Consequently, the main goal to prove the valorization of depolymerized cutin obtained from agro-food wastes for the plasticization of PLA was achieved for the first time and this work paves the way for the use of cutin fatty acids as bio-based plasticizers for various bioplastics.

## Figures and Tables

**Table 1:** Glass transition temperatures (T_g_) and peak melting temperatures (T_m_) of the cutin monomer and derivatives (**1**–**4**).

Entry	Monomer	T_g_ (°C)	T_m_ (°C)
1	1	−45	59
2	2	−34	12
3	3	−59	−15
4	4	−61	−10

**Table 2:** Hansen solubility parameters, $\delta_{t}$ and their dispersive, $\delta_{d}$ polar, $\delta_{p}$ and hydrogen bonding group $\delta_{h}$ contributions for derivatives **1**–**4** and PLA.

Entry	*δ* (J/cm^3^)^1/2^	10,16-diHHDA (1)	10,16-diHHDME (2)	10,16-diAHDA (3)	10,16-diAHDME (4)	PLA *
1	$\delta_{d}$	17.3	17.1	17.5	17.3	19.8
2	$\delta_{p}$	3.9	3.7	3.1	4.0	4.0
3	$\delta_{h}$	13.4	12.6	8.4	7.6	6.7
4	$\delta_{t}$	22.2	21.6	19.7	19.3	22.0

* The solubility parameters components considered for PLA were those determined in the literature by inverse gas chromatography technique [37].

**Table 3:** Glass transition temperature (T_g_), peak melting temperature (T_m_), and crystallinity degree corresponding to the different mixtures of PLA/cutin derivatives (**1**–**4**).

Entry	Mixture	[Monomer] (% *w*/*w*)	T_g_ (°C)	T_m_ (°C)	Crystallinity (%)
1	PLA	//	64	158	42
2	PLA−1−10	1, 10	61	153	32
3	PLA−1−20	1, 20	59	152	30
4	PLA−2−20	2, 20	59	149	41
5	PLA−2−30	2, 30	57	148	47
6	PLA−2−40	2, 40	50	149	44
7	PLA−3−20	3, 20	47	151	44
8	PLA−3−30	3, 30	39	150	29
9	PLA−3−40	3, 40	49	152	49
10	PLA−4−20	4, 20	49	151	45
11	PLA−4−30	4, 30	36	150	45
12	PLA−4−40	4, 40	34	150	48

## Data Availability

Publicly available datasets were analyzed in this study. This data can be found here: https://github.com/raffasuri/CutToPro-project.git, last accessed on 11 March 2023.

## Appendix A

The acetylation reaction was performed under different conditions to find a protocol able to give a high yield (Table A1). Hydrochloric acid, sulfuric acid, and trifluoroacetic acid were tested at 60 °C (**entries 1**–**2**): when sulfuric acid was employed the reaction mixture appeared as a black suspension and signs of degradation of the substrate were detected by ^1^H-NMR analysis after 1 h. In light of this result, HCl was chosen as a catalyst. Both diluted and concentrated acid can promote the reaction to give the product in medium to high yield (**entries 3**–**4**). To limit the amount of water in the reaction mixture, and the subsequent degradation of Ac_2_O, concentrated HCl was preferred (**entry 7**).

**Table A1:** Reaction conditions tested to obtain the molecule **3**.

Entry	[1] (M)	Catalyst (µL)	t (h)	T (°C)	Yield (%)
1	0.8	HCl 1 M (50)	3	60	65 ^1^
2	0.8	H_2_SO_4_ conc. (50)	1	60	58 ^1,3^
3	0.5	HCl 1 M. (50)	3	80	76 ^1^
4	0.5	HCl conc. (50)	3	80	73 ^1^
5	1.5	HCl conc. (25)	3	90	78 ^1^
6	0.3	HCl conc. (25)	3	90	87 ^1^ 80 ^2^
7	0.3	HCl conc. (25)	3	100	90 ^1^ 88 ^2^

^1^ analytical yield; ^2^ isolated yield; ^3^ degradation.

Concerning the molar volumes for the calculation of the dispersive, polar, and hydrogen bonding solubility parameter components ($\delta_{d}$, $\delta_{p},$ $\delta_{h}$) (Equations (2)–(5)), the following equation was used:

(A1) $$V=\Sigma (n*V_{i})$$

where n is the number of functional groups of the same type present in the chemical structure under investigation and $V_{i}$ is the molar volume for each functional group *i* shown in Table A2 for Hoy’s system for the molar attraction function [36].

**Table A2:** The dispersion components $F_{di}$, and the polar components $F_{pi}$ of the molar attraction constant as well as the hydrogen bonding energies $E_{hi}^{}$ for each functional group, *i* present in the literature [36].

Functional Group	*F_di_* (J^1/2^cm^3/2^/mol)	*F_pi_* (J^1/2^cm^3/2^/mol)	*E_hi_* (J/mol)	$V_{i}$ (cm^3^/mol)
-OH	210	500	20,000	12.45
-COOH	530	420	10,000	26.1
-CH_2_-	270	0	0	15.55
-CH-	80	0	0	9.56
-CH_3_	420	0	0	21.55
-COO-	390	490	7000	23.7

Regarding the DSC curves obtained when analyzing the mixtures PLA−X−Y reported in Table 3, the regions relative to the melting point peaks during the first heating step are reported in Figure A2.
